# Effect of Tetrandrine against *Candida albicans* Biofilms

**DOI:** 10.1371/journal.pone.0079671

**Published:** 2013-11-18

**Authors:** Lan-Xue Zhao, De-Dong Li, Dan-Dan Hu, Gan-Hai Hu, Lan Yan, Yan Wang, Yuan-Ying Jiang

**Affiliations:** 1 New Drug Research and Development Center, School of Pharmacy, Second Military Medical University, Shanghai, China; 2 Department of Pharmacy, Institute of Medical Sciences, Shanghai Jiaotong University School of Medicine, Shanghai, China; 3 Department of Pharmacy, Fujian University of Traditional Chinese Medicine, Fuzhou, China; Ghent University, Belgium

## Abstract

*Candida albicans* is the most common human fungal pathogen and has a high propensity to develop biofilms that are resistant to traditional antifungal agents. In this study, we investigated the effect of tetrandrine (TET) on growth, biofilm formation and yeast-to-hypha transition of *C. albicans*. We characterized the inhibitory effect of TET on hyphal growth and addressed its possible mechanism of action. Treatment of TET at a low concentration without affecting fungal growth inhibited hyphal growth in both liquid and solid Spider media. Real-time RT-PCR revealed that TET down-regulated the expression of hypha-specific genes *ECE1*, *ALS3* and *HWP1*, and abrogated the induction of *EFG1* and *RAS1*, regulators of hyphal growth. Addition of cAMP restored the normal phenotype of the SC5314 strain. These results indicate that TET may inhibit hyphal growth through the Ras1p-cAMP-PKA pathway. *In vivo*, at a range of concentrations from 4 mg/L to 32 mg/L, TET prolonged the survival of *C. albicans-*infected *Caenorhabditis elegans* significantly. This study provides useful information for the development of new strategies to reduce the incidence of *C. albicans* biofilm-associated infections.

## Introduction

*Candida albicans* is the most common fungal pathogen and may cause life-threatening invasive infections, especially in immunocompromised individuals [1], [2]. Antifungal agents available are limited in clinic, and drug resistance has become a significant threat [3], [4]. *C. albicans* has a high propensity to develop biofilms on the surfaces of almost any medical devices, such as stents, shunts, prostheses, implants, endotracheal tubes, pacemakers and various types of catheters [5], resulting in biofilm-associated infections [6]–[8]. More specifically, it is the fourth leading cause of vascular catheter-related infections and the third leading cause of urinary catheter-related infections [9]–[12]. Among vascular catheter-related infections, those due to *Candida* spp. are associated with the highest rate of mortality [9], [13], [14]. The *C. albicans* biofilms are structured microbial communities with *C. albicans* cells embedded in a matrix of extracellular polymeric substances produced by the cells [15]–[18]. Comparing to planktonic cells, *C. albicans* cells in biofilms display severe resistance to a wide variety of clinical antifungal agents, including amphotericin B and fluconazole [19]–[22]. There is an urgent need to develop new antifungal agents against *C. albicans* biofilms.

**Figure 1:**
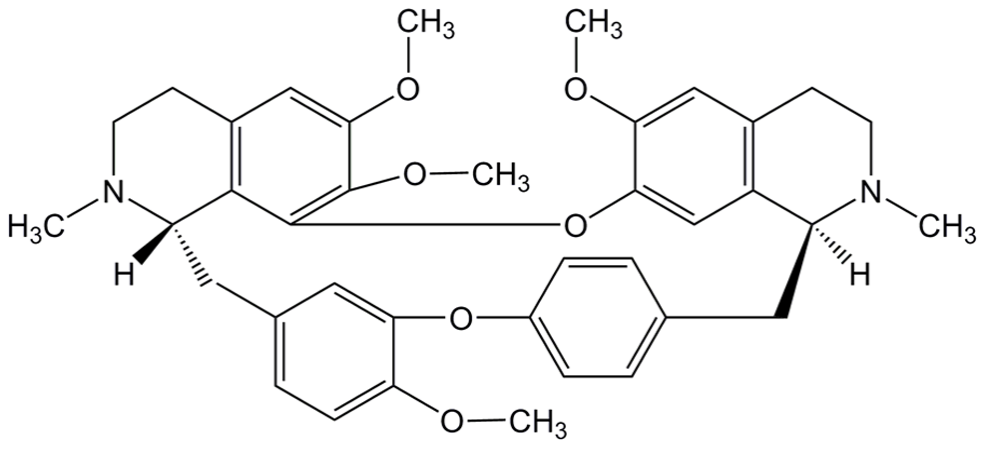
Chemical structure of TET.

Tetrandrine (TET) (Fig. 1) is a bis-benzylisoquinoline alkaloid compound originating from several natural plant sources, including *Stephania tetrandra* [23], [24]. This alkaloid displays low toxicity [25] and has been used in China for the treatment of angina, hypertension, silicosis and arthritis [26]–[30]. Besides, TET could reduce acute radiation injury [31], [32] and exhibited anti-inflammatory [32]–[34] and anti-tumor [35], [36], [37], [38] activities. In more details, TET was reported to block voltage-gated Ca^2+^ channels in mammalian cells [38], inhibit NF-κB activation in the alveolar macrophage [33], induce apoptosis and growth arrest in human leukemic HL-60 cells and lung carcinoma cells [36], [37], serve as a MDR (multidrug drug resistance) modulator for the treatment of P-glycoprotein-mediated MDR cancers [35]. Interestingly, it exhibited synergistic effect with ketoconazole against drug resistant *C. albicans* [39] and synergism with econazole against *Trichophyton mentagrophytes* [40]. Nevertheless, its activity against *C. albicans* biofilms has not yet been investigated.

In this study, we evaluated the activity of TET against *C. albicans* biofilms, and revealed that the anti-biofilm activity of TET was associated with Ras/cAMP pathway.

## Results

### TET inhibits the formation of *C. albicans* biofilms *in vitro*

**Figure 2:**
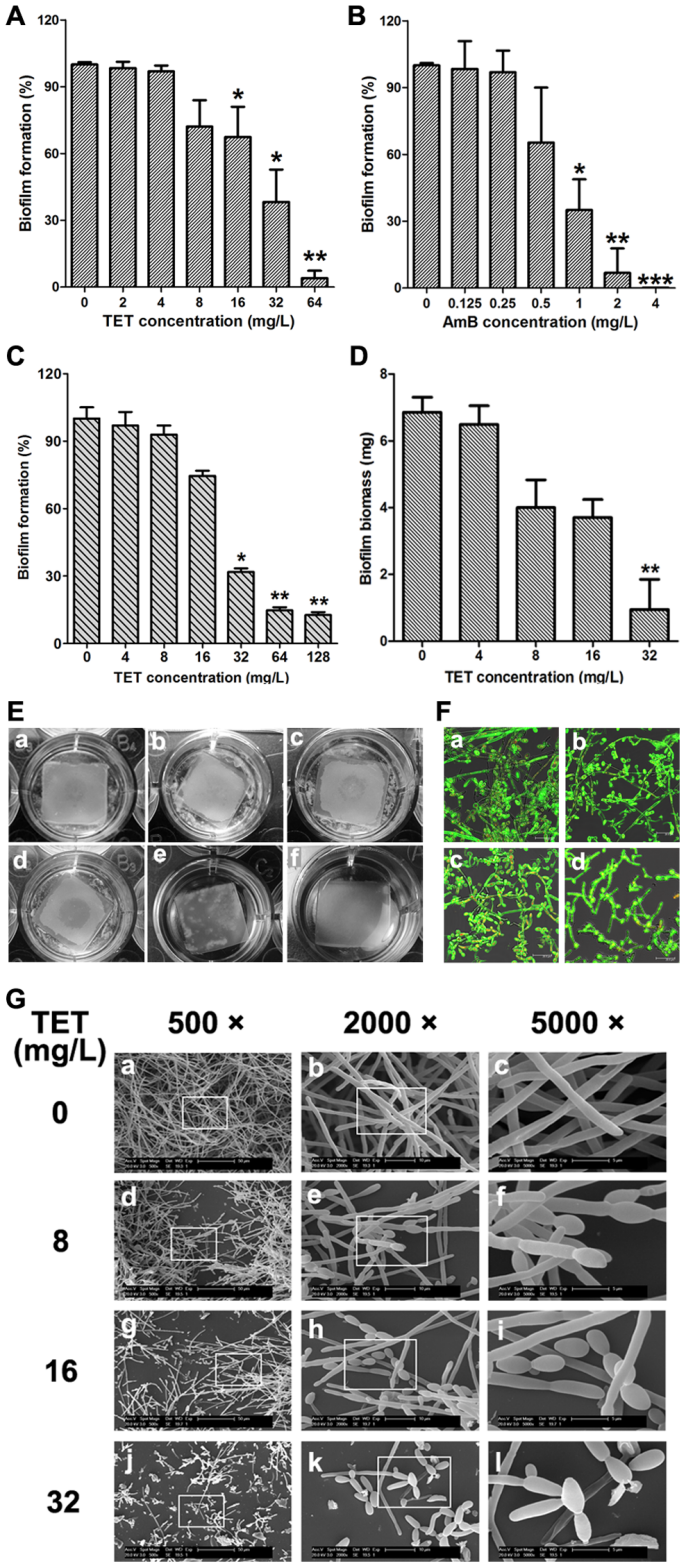
TET inhibits *C. albicans* SC5314 biofilm formation *in vitro*. (A) Effects of different concentrations of TET on biofilm formation. (B) Effects of different concentrations of Amphotericin B on biofilm formation. AmB: amphotericin B. (C) Effects of different concentrations of TET on the maintenance of mature biofilms. Biofilm formation was evaluated by XTT reduction assay, and the results were presented as the percentage compared to the control biofilms formed without TET treatment. Biofilm formation results represent the mean ± standard deviation for five independent experiments. * *P*<0.05 compared to the control biofilms, ** *P*<0.01 compared to the control biofilms. (D) Effects of different concentrations of TET on biofilms formed on silicone pads. Standard deviations are depicted and based on 6 silicone pad measurements. ** *P*<0.01. (E) Screen for TET-treated biofilms formed on silicone pads. The wells are shown for a: normal biofilm. b: cells were treated with 4 mg/L TET. c: 8 mg/L TET. d: 16 mg/L TET. e: 32 mg/L TET. f: uninoculated control. (F) Effects of different concentrations of TET on biofilm formation presented by using CLSM. a: Control. b: 8 mg/L of TET. c: 16 mg/L TET. d: 32 mg/L TET. (G) Effects of different concentrations of TET on biofilm formation presented by using SEM. The inset in the 500 ×, 2000× panels show the area that was magnificated.

The effect of TET on *C. albicans* biofilm formation was evaluated by XTT reduction assay (Fig. 2). It was found that addition of TET to *C. albicans* cells after 90-min adhesion inhibited biofilm formation in a dose-dependent manner (Fig. 2A). More specifically, 16 mg/L TET inhibited biofilm formation significantly (*P*<0.05), and this anti-biofilm effect increased with increasing TET concentrations. In the 64 mg/L TET group, the biofilm formation was less than 5% as compared with the control group without TET treatment. Notably, under the condition that the biofilms were mature after 24 h incubation at 37°C, TET also inhibited biofilms in a dose-dependent manner (Fig. 2C). 32 mg/L TET inhibited mature biofilms significantly (*P*<0.05). The anti-biofilm effect increased with increasing TET concentrations. In the 64 mg/L TET group, the maintenance of biofilm architecture was about 15% as compared with the control group without TET treatment. Collectively, TET showed a significant anti-biofilm effect on both developing biofilms and mature biofilms.

The result of biofilm biomass determination confirmed the dose-dependent anti-biofilm effect of TET, and 32 mg/L TET inhibited biofilm formation significantly (P<0.01; Fig. 2D). Accordingly, the anti-biofilm effect of TET could be observed visually (Fig. 2E). Compared with the TET-free control biofilms on silicone pads (Fig. 2Ea), biofilms in the 4, 8 mg/L TET group (Fig. 2Eb, c) were thinner and incomplete. With increasing TET concentrations, the effect of TET on biofilm formation became more obvious. In the 32 mg/L TET group (Fig. 2Ee), the silicone pad was maintained clean, indicating that the biofilm formation was disrupted completely.

The anti-biofilm effect of TET was further confirmed by confocal laser scanning microscopy (CLSM, Fig. 2F) and scanning electron microscopy (SEM, Fig. 2G). Compared with the normal thick biofilm with true hyphae criss-crossing (Fig. 2Fa, 2Ga-c), *C. albicans* biofilm formation was disrupted by TET in a dose-dependent manner. 8 mg/L of TET (Fig. 2Fb, 2Gd-f) led to the reduction in cell density and defect in filamentation. With increasing the TET concentration to 16 mg/L and 32 mg/L (Fig. 2Fc-d, 2Gg-l), cell density was further reduced and the defect in filamentation became more obvious.

**Figure 3:**
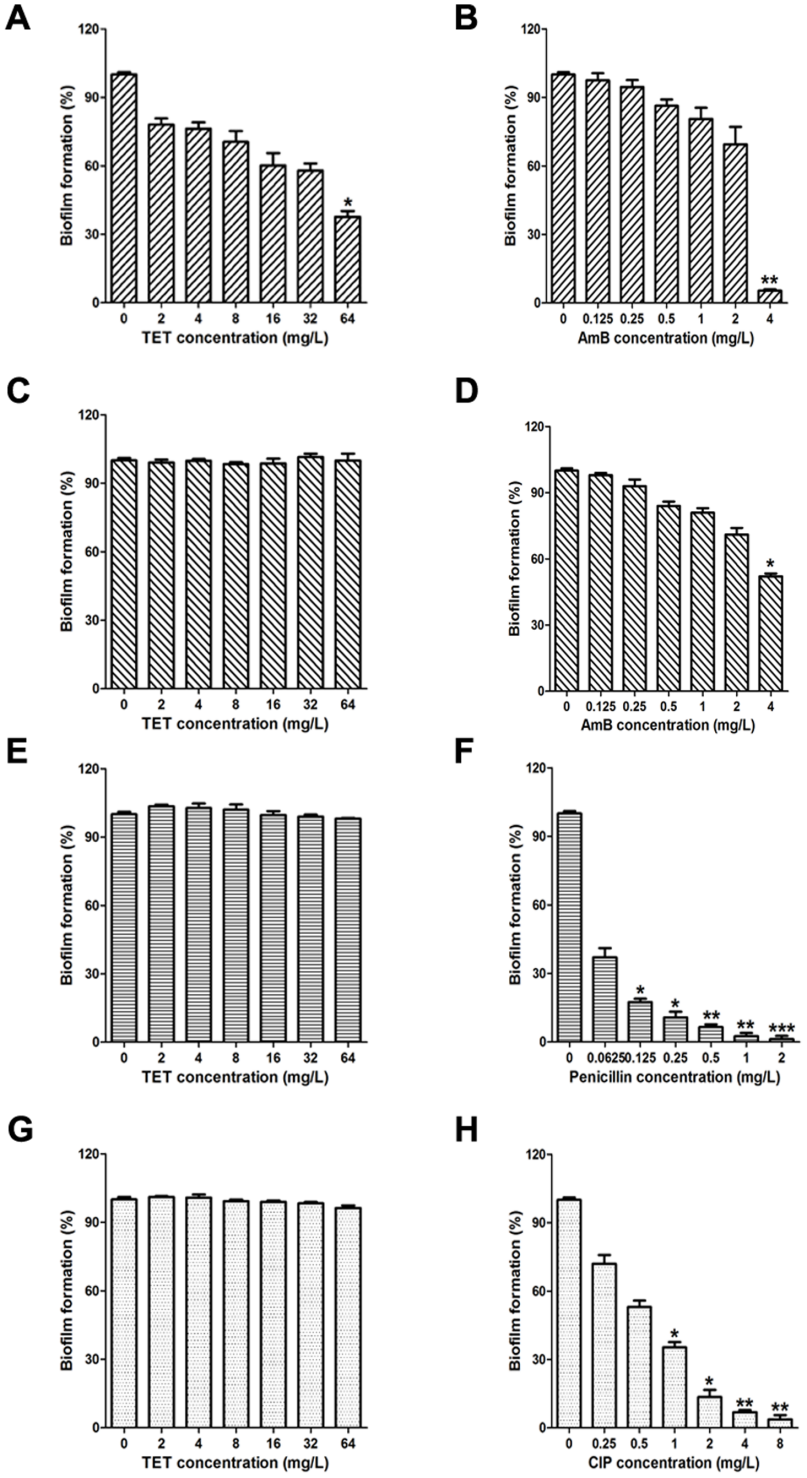
Effects of TET on fungal and bacterial biofilm formation *in vitro*. (A) TET against *C. neoformans* H99; (B) Amphotericin B against *C. neoformans* H99, AmB: amphotericin B; (C) TET against *A. fumigatus* T308073458; (D) Amphotericin B against *A. fumigatus* T308073458, AmB: amphotericin B; (E) TET against *S. aureus*; (F) Penicillin against *S. aureus*; (G) TET against *P. aeruginosa*; (H) Ciprofloxacin against *P. aeruginosa*, CIP: ciprofloxacin. * *P*<0.05 compared to the treatment-free control biofilm, ** *P*<0.01 compared to the treatment-free control biofilm, *** *P*<0.001 compared to the treatment-free control biofilm.

We further evaluated the activity of TET against biofilms of other fungi and bacteria (Fig. 3). *Cryptococcus neoformans* strain H99, *Aspergillus fumigatus* strain T308073458, *Staphylococcus aureus* strain Newman and *Pseudomonas aeruginosa* strain PA14 were used in this study. TET exhibited weak anti-biofilm effect against *C. neoformans*: it inhibited the biofilms significantly only when the drug concentration was as high as 64 mg/L (Fig. 3A). No anti-biofilm effect was observed of TET against *A. fumigatus*, *S. aureus* and *P. aeruginosa*, even under the condition that the concentration was high as 64 mg/L (Fig. 3C, E, G). Collectively, the strong anti-biofilm effect of TET was selective against *C. albicans*.

### TET decreases cellular surface hydrophobicity (CSH) of *C. albicans* biofilm

**Figure 4:**
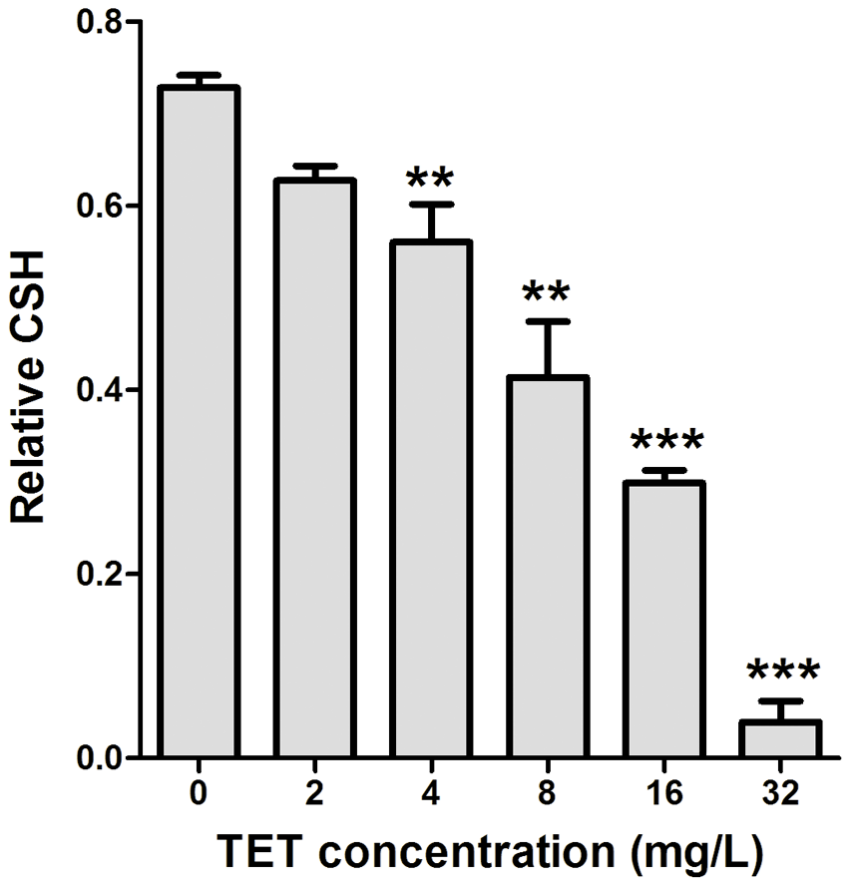
Effects of different concentrations of TET on CSH of *C. albicans* SC5314. CSH was estimated by using the water-hydrocarbon two-phase assay. Standard deviations are depicted and based on three independent experiments. ** *P*<0.01, *** *P*<0.001.

Knowing that there is a positive correlation between CSH and adhesion of *C. albicans* [41]-[43], we examined the effect of TET on CSH. The normal CSH of *C. albicans* was shown as 0.73 in this work. Our results showed that 4 mg/L TET significantly decreased CSH to 0.56 (*P*<0.01; Fig. 4). In addition, TET decreased CSH of *C. albicans* biofilm in a dose-dependent manner, and it decreased to 0.04 in the 32 mg/L TET group (Fig. 4).

### TET retards growth of *C. albicans*

**Figure 5:**
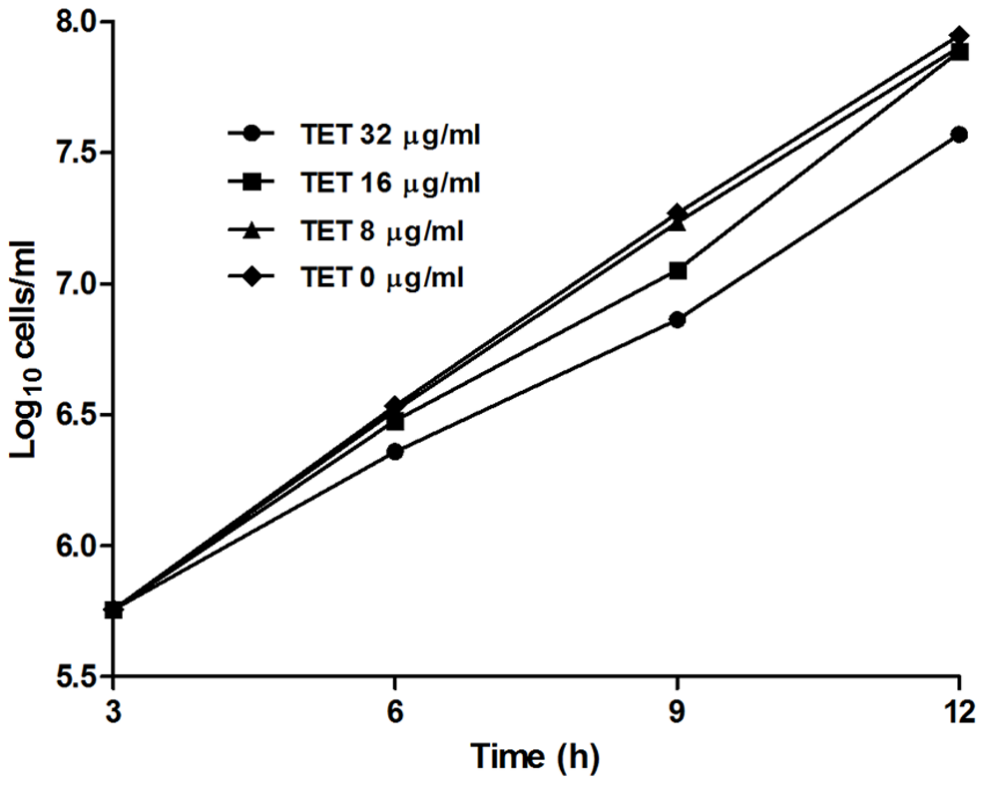
Time-growth curves of different concentrations of TET on *C. albicans* strain SC5314.

The effect of TET on growth of *C. albicans* was further investigated. Time-growth curves indicated that 8 and 16 mg/L TET could not affect the growth of *C. albicans* significantly, where the cell density reached 1×10^8^ cells/ml after 12-h culture, which was similar to that in the control group without TET treatment (Fig. 5). At 32 mg/L, TET slowed down the growth of *C. albicans*, and the cell density were 3.7×10^7^ cells/ml after 12 h culture (Fig. 5).

We also carried out a standard antifungal susceptibility test to investigate the activity of TET on growth of *C. albicans*. Besides the normally used *C. albicans* strain SC5314, another fluconazole-susceptible strains Y0109 and two fluconazole-resistant strains, 0304103 and 01010, were used in this experiment. MIC_50_ was determined as the lowest concentration of the drug that inhibited fungal growth by 50%. TET exhibited weak antifungal activity: the MIC_50_s against both the fluconazole-susceptible strains and the fluconazole-resistant strains were 32 mg/L (Table 1), and the MIC_80_s of TET against all the four strains tested were 64 mg/L.

**Table 1:** The MIC_50_ of TET and fluconazole against *C. albicans* strains.


Strains	MIC_50_ (mg/L)	
	TET	Fluconazole
Fluconazole-susceptible strains		
SC5314	32	0.125
Y0109	32	0.25
Fluconazole-resistant strains		
304103	32	>64
1010	32	>64

### TET inhibits hyphal formation of *C. albicans*

**Figure 6:**
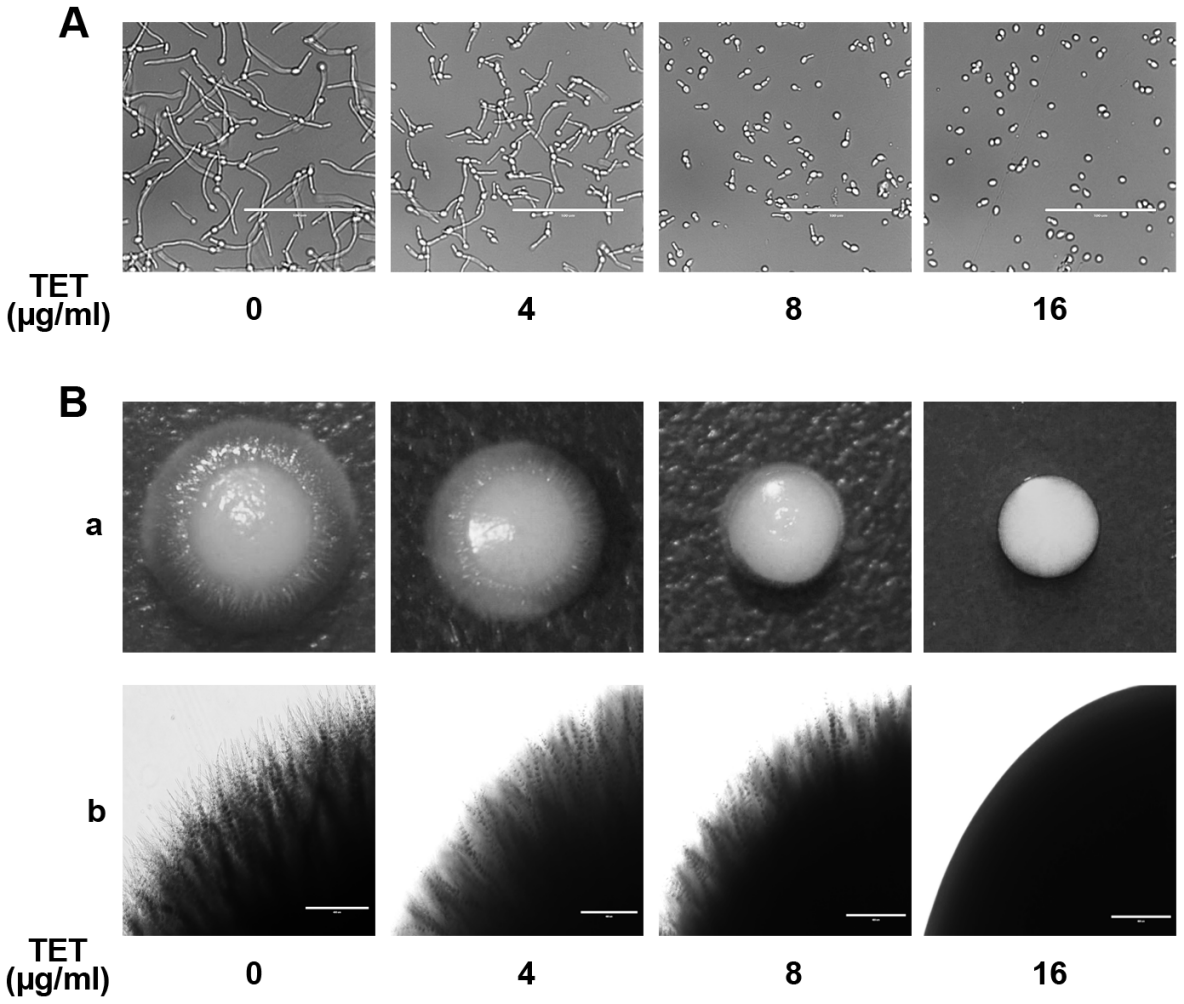
Effects of different concentrations of TET on hyphal formation in Spider medium. (A) Log phase cells were incubated in liquid Spider medium at 37°C. Cells were photographed after 4 h of incubation in Spider medium. Observed with a inverted phase contrast microscope (AMG® EVOS xl) with a×40 objective. (B) Approximately 10 cells were plated on Spider solid medium. Incubation time and temperature were 5 d at 37°C.

To study the effect of TET on yeast-to-hypha morphological transition of *C. albicans*, *C. albicans* cells were grown in liquid Spider medium known to induce morphological transition. In TET free Spider medium, *C. albicans* cells formed true hyphae (Fig. 6A). At 4 mg/L, TET inhibited the yeast-to-hypha morphological transition to some extent, and the inhibition occurred in a dose-dependent manner. The addition of 16 mg/L TET totally disrupted the hyphal formation (Fig. 6A). In accordance, the inhibition effect of TET on hyphal formation was also observed on solid Spider medium (Fig. 6B). More specifically, at 4 mg/L, TET inhibited the developing of radial colonies to some extent (Radial colonies usually indicate mycelial cells inside the colonies while smooth colonies indicate budding yeast cells inside[44]), and in 16 mg/L TET group, only smooth-edged colonies were observed (Fig. 6B). Collectively, TET inhibited the yeast-to-hypha morphological transition in a dose-dependent manner in Spider medium.

### Exposure to TET alters *C. albicans* gene expression

**Figure 7:**
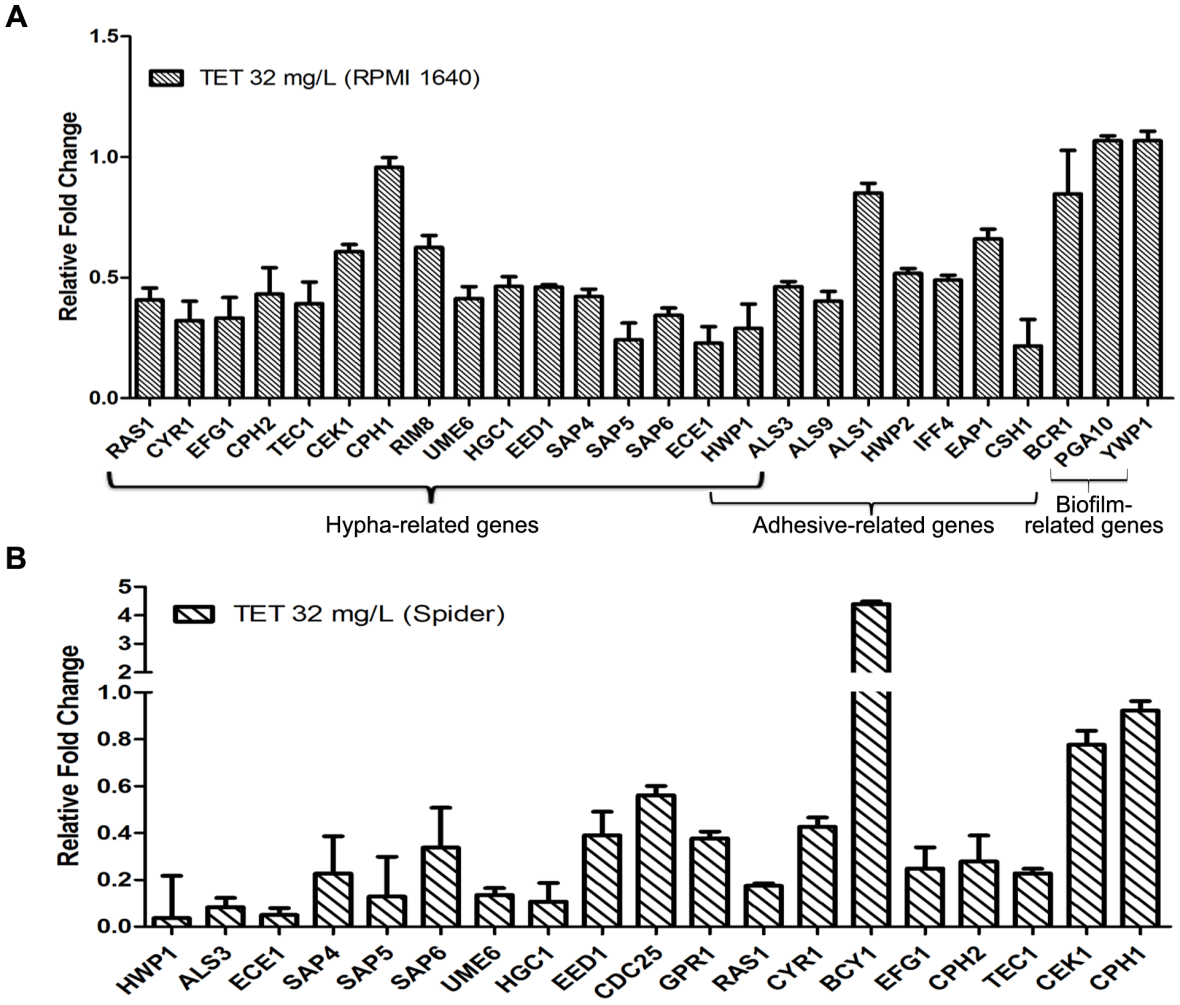
Gene expression changes of some important biofilm formation related genes. The *C. albicans* strain tested was SC5314. The concentration of TET was 32 mg/L. All genes were examined by real-time RT-PCR with gene-specific primers. Gene expression was indicated as a fold change relative to that of the control group treated with DMSO. (A) in RPMI 1640 medium. (B) in Spider medium. Data are shown as mean ± SD from three experiments.

To understand the anti-biofilm mechanism of TET, we further investigated the expression changes of the known adhesion-related, hypha-related and biofilm-related genes after TET treatment using real-time RT-PCR. In RPMI 1640 medium at 37°C, the hypha-specific genes such as *ECE1*, *HWP1*, *ALS3*, *SAP4*, *SAP5*, *SAP6*, *UME6*, *EED1* and *HGC1* were down-regulated after 32 mg/L TET treatment (Fig. 7A). Some regulation genes, including *RAS1*, *CYR1*, *EFG1*, *CPH2*, and *TEC1* were also down-regulated. Moreover, adhesion-specific genes *CSH1*, *IFF4* and *ALS9* were down-regulated by 0.22, 0.49 and 0.40 fold respectively. Nevertheless, *ALS1*, *EAP1* and *HWP2*, three critical adhesion-related genes were not affected significantly after 32 mg/L TET treatment. Taken together, the real-time RT-PCR results indicated that TET treatment down-regulated the expression of some hypha-specific genes and some genes known to regulate the yeast-to-hypha transition in RPMI 1640 medium.

Similar results were obtained in Spider at 37°C (Fig. 7B). Hypha-specific genes *HWP1*, *ALS3* and *ECE1* were down-regulated with the relative fold being 0.036, 0.083 and 0.050 respectively. Besides, *SAP4*, *SAP5*, *SAP6*, *UME6*, *HGC1* and *EED1* were also down-regulated. Some regulation genes including *RAS1*, *CYR1*, *EFG1*, *CPH2* and *TEC1* were down-regulated as well.

### Exogenous cAMP reverts the morphogenesis defect caused by TET

**Figure 8:**
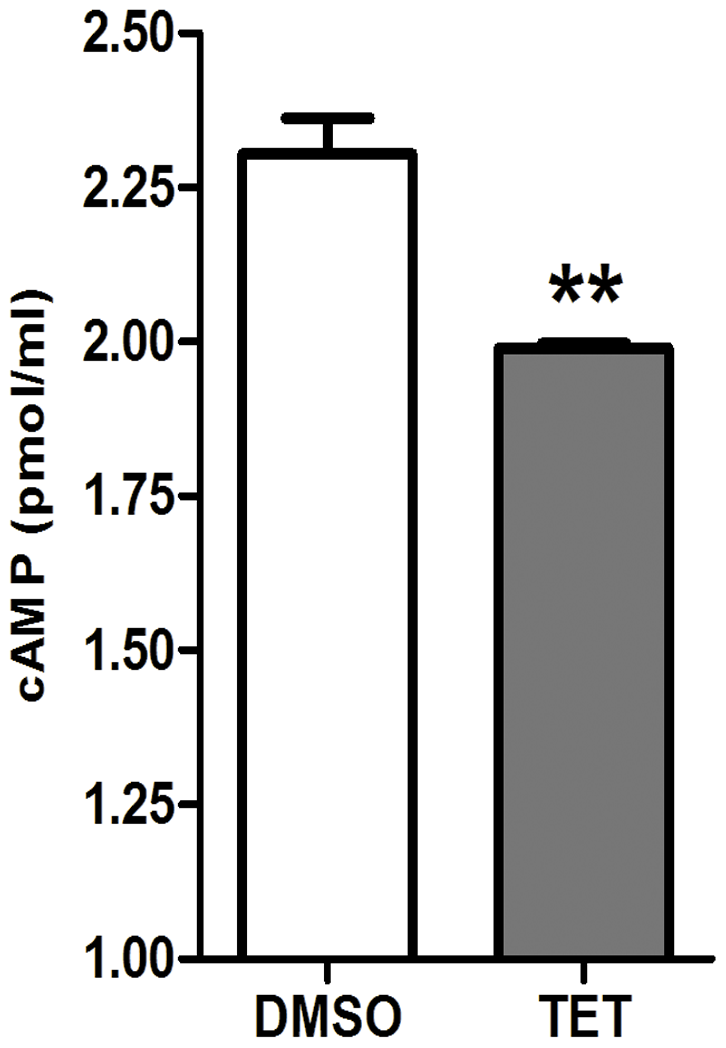
Determination of intracellular cAMP level. Exponentially growing *C. albicans* SC5314 cells incubated at 37°C in Spider medium in the presence of 32 mg/L TET and harvested at the 60 min time point. The cAMP content was measured using the cAMP Enzyme Immunoassay Kit according to the manufacturer’s instructions. ** *P*<0.01.

**Figure 9:**
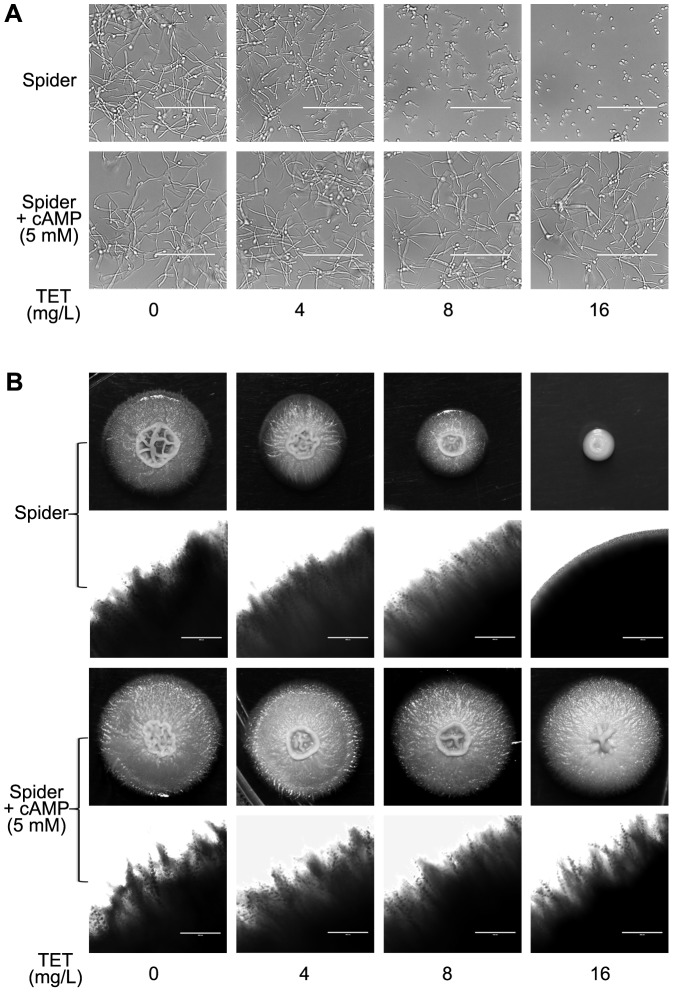
Addition of exogenous cAMP reverts the morphological transition defect of *C. albicans* SC5314 caused by TET. Exponentially growing *C. albicans* SC5314 cells were transferred to Spider medium. (A) Cells were incubated in liquid Spider medium supplemented without or with cAMP (final concentration 5 mM). The cells were incubated at 37°C for 4 h. Magnification 40 ×. (B) Hyphal formation on solid Spider medium plate with the same concentrations of cAMP and TET as in liquid Spider medium.

Since a series of important genes in Ras/cAMP pathway, including *RAS1*, *CYR1*, *EFG1*, *CPH2*, *TEC1*, *BCY1*, *ECE1, ALS3*, *HWP1* and *HGC1* [45], [46] were down-regulated after TET treatment, cAMP levels were measured in *C. albicans* cells. A significant decrease of cAMP level was observed in the 32 mg/L TET-treated cells (*P*<0.01; Fig. 8). Interestingly, exogenous cAMP reverted the morphogenesis defect caused by TET (Fig. 9). More specifically, with the addition of 5 mM cAMP in TET treated cultures, true hyphae were observed both in liquid and on solid Spider media (Fig. 9).

### TET exhibits antifungal activity *in vivo* in a *Caenorhabditis elegans* infection model

**Figure 10:**
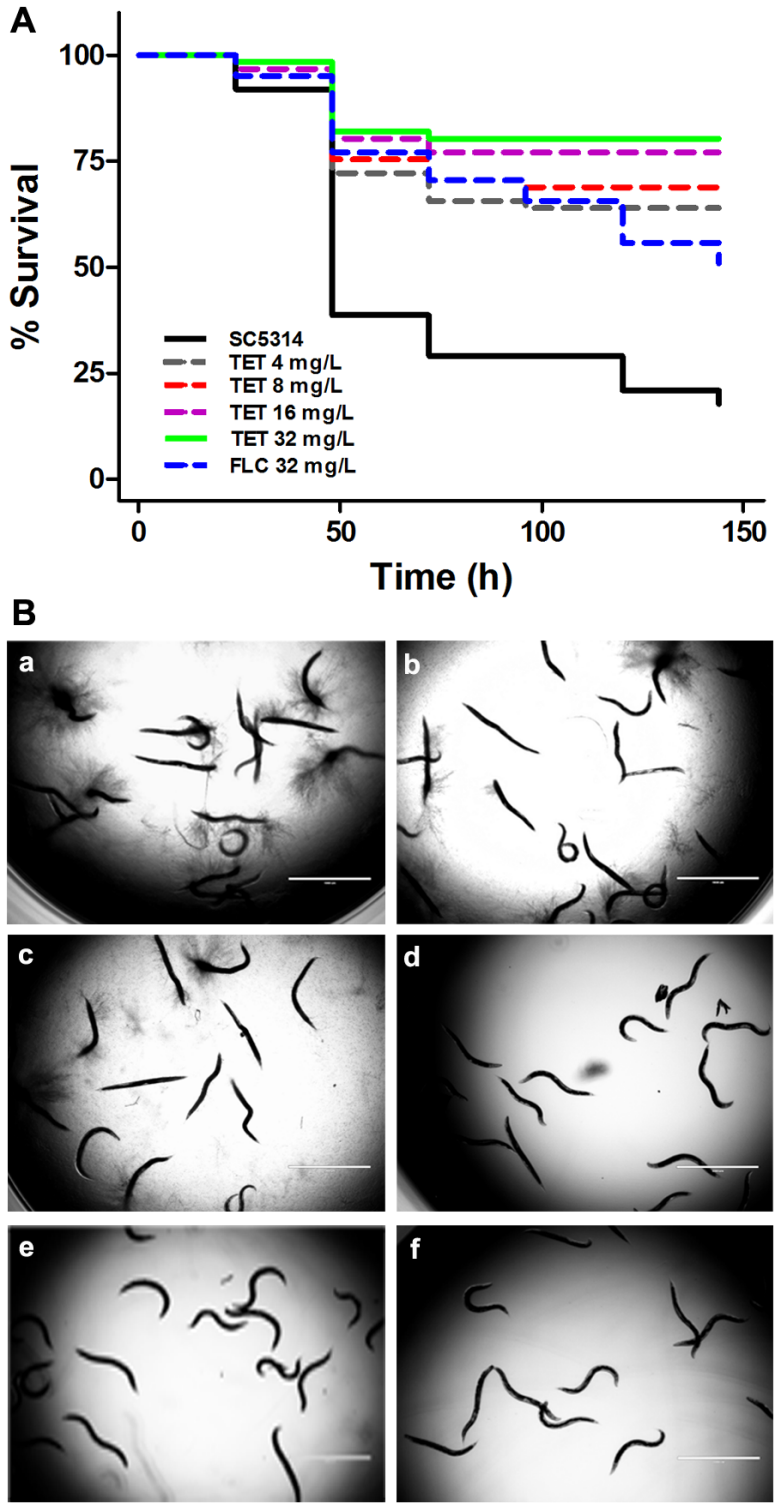
TET prolongs the survival of *C. elegans glp-4; sek-1* nematodes infected by *C. albicans* SC5314. (A) Nematodes were infected with *C. albicans* for 4 h and then moved to pathogen-free liquid medium in the presence of TET (4 mg/L, 8 mg/L, 16 mg/L, 32 mg/L, *P*<0.0001), FLC (32 mg/L, *P*<0.0001) or DMSO. Dead worms were counted and removed daily. (B) After exposure to strain *C. albicans* SC5314, *C. elegans* nematodes were piped into 12-well plates that contain TET or DMSO. TET exhibited antifungal activity. a: Treatment free control group (DMSO added); b: TET 4 mg/L; c: TET 8 mg/L; d: TET 16 mg/L; e: TET 32 mg/L; f: FLC 32 mg/L.

Since TET inhibited yeast-to-hypha morphological transition, the most widely acknowledged pathogenic trait of *C. albicans*, we further investigated the *in vivo* antifungal activity of TET using a *C. elegans*–*C. albicans* infection model. At a range of concentrations, from 4 mg/L to 32 mg/L, TET significantly protected *C. elegans* from *C. albicans* infection (*P*<0.0001; Fig. 10A). More specifically, we found that under the conditions used in this study more than half of the worms died within the first 48 h after infection with *C. albicans* strain SC5314 (Fig. 10A), and every dead worm had visible hyphae piercing the cuticle (Fig. 10Ba). At 144 h after infection, less than 20% of the worms were alive (Fig. 10A). In contrast, with 4 mg/L to 32 mg/L TET treatment, > 60% of the worms were alive until the end of 144 h of our observation period (Fig. 10A), and little even no hyphae were observed on the living worms (Fig. 10Bb-e).

**Figure 11:**
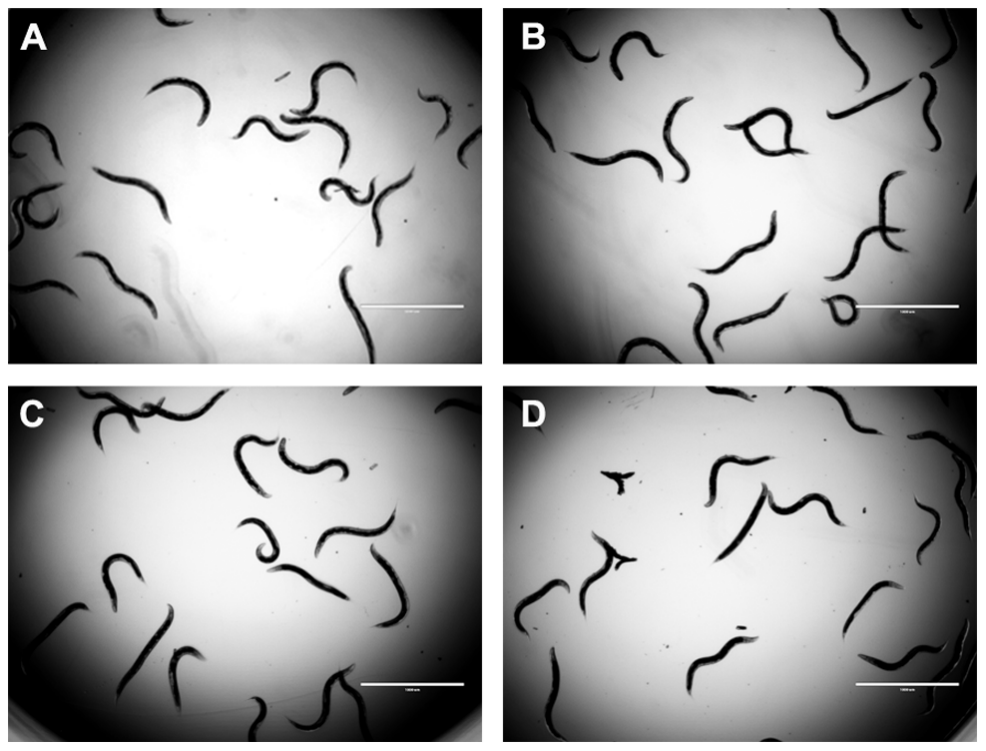
TET shows no toxicity on uninfected *C. elegans glp-4; sek-1* nematodes. *C. elegans glp-4; sek-1* nematodes were pipetted into 12-well plates that contained different concentrations of TET, incubated at 25°C, and observed daily. On day 2, the worms were photographed. (A) 32 mg/L TET; (B) 64 mg/L TET; (C) 128 mg/L TET; (D) Treatment free group with the solvent DMSO added.

We further tested the toxicity of TET using uninfected adult *C. elegans* worms. Final concentrations at 32 mg/L, 64 mg/L and 128 mg/L were used respectively. At all the concentrations tested, no toxicity of TET was observed, and the worms all looked healthy. There was no difference between the TET treatment groups and the drug-free group (Fig. 11).

## Discussion

*C. albicans* biofilms constitute a threat to successful antifungal treatment [47]. In this study, we revealed the anti-biofilm activity of TET against *C. albicans*. TET exhibited significant effect against both biofilm formation and maintenance of mature biofilms *in vitro*. Interestingly, the compound exhibited satisfactory anti-biofilm activity selectively against *C. albicans*. TET showed only weak anti-biofilm activity against *C. neoformans* and no effect against *A. fumigatus*, *S. aureus* and *P. aeruginosa*, at least at the concentrations we tested. We further investigated the mechanism of TET against *C. albicans* biofilms and found that TET could decrease CSH, retard the growth of *C. albicans* at high concentrations, and suppress the yeast-to-hypha morphological transition. The results of real-time RT-PCR indicated that some important filamentation genes were differentially expressed after exposure to TET. We further revealed that the effect of TET was related to Ras/cAMP pathway, and exogenous cAMP could restore the morphological transition of *C. albicans* under the condition of TET exposure. Finally, we revealed the antifungal activity of TET *in vivo* using the *C. elegans-C. albicans* infection model.

To our knowledge, this is the first report indicating that TET had a significant anti-biofilm effect against *C. albicans*. Notably, TET could not only inhibit the formation of biofilms but also destroy the maintenance of mature biofilms. More specifically, we revealed that the MIC_50_ of TET against *C. albicans* SC5314 was 32 mg/L. 32 mg/L (1×MIC) of TET inhibited the development of more than 60% biofilms, and destroyed the maintenance of about 60% mature biofilms. The effect of TET was good compared with various other antifungal agents. Vila *et al* [48] found that at the concentration of 16×MIC, fluconazole could not inhibit the development of biofilms, and that amphotericin B at the concentration of 1×MIC could inhibit the development of biofilms but could not affect the maintenance of mature biofilms. Collectively, TET possesses satisfactory anti-biofilm activity.

Our data indicate that TET could inhibit biofilm formation through decreasing adhesion and morphological transition, rather than inhibiting the growth of *C. albicans*. Adhesion to biomaterial surfaces, growth of cells to form an anchoring layer, and morphological transition to form a complex three-dimensional structure are known to be three stages for biofilm formation [49]-[53]. Our data indicate that 16 mg/L (1/2×MIC_50_) TET significantly inhibited biofilm formation, severely decreased CSH (indicating adhesion ability [43]), and obviously inhibited the yeast-to-hypha morphological transition in both liquid and solid Spider medium, while 16 mg/L TET had no significant influence on the growth of *C. albicans*. Thus, the anti-biofilm effect of TET seems attributable to its anti-adhesion and anti-morphological-transition activities.

Our real-time RT-PCR data indicate that some important hypha and adhesion-related genes, including *ECE1, HGC1*, *RAS1*, *CYR1*, *EFG1*, *CPH2*, *TEC1*, *HWP1* and *ALS3* were down-regulated after 32 mg/L TET treatment. *ECE1* is a hypha-specific gene and its expression correlates with the extent of hyphal cell elongation [54]. *HGC1* encodes a cyclin partner and functions in maintaining hyphal growth [55]. Ras1p is a GTPase that plays roles in inducing hyphal formation by activating both Ras/cAMP pathway and MAPK [56], [57] pathway. Cyr1 integrates environmental signals from a range of sources and is essential for hyphal formation [58]. Efg1 [59], Cph2 [60] and Tec1 [61] are transcription factors that positively regulate the expression of hypha-specific genes. More specifically, Efg1 is a transcription factor of the Ras/cAMP pathway, which plays important roles in regulating the expression of some hypha-specific genes, including *ECE1*, *HWP1* and *ALS3* [62]. Accordingly, *ECE1*, *HWP1* and *ALS3* were all down-regulated after TET treatment. Besides the hypha-specific character, *HWP1* is also a unique adhesion gene expressing on the hyphal surface. Biofilms lacking *HWP1* gene were prone to detach from the abiotic substrate [63], [64]. *ALS3* is an *ALS* family gene that plays an essential role in the adherence stage of *C. albicans* [65], [66]. The down-regulation of these genes may contribute to the hyphal formation and adhesion defect of *C. albicans* after TET treatment.

Interestingly, many down-regulated genes after TET treatment, including *HWP1*, *ALS3*, *ECE1* and *HGC1*, were genes regulated by Ras/cAMP pathway [45], [46]. Thus we speculated that the anti-biofilm effect of TET might be related to the down-regulation of Ras/cAMP pathway. To verify this hypothesis, we determined the cAMP level and revealed a significant decrease in cAMP level after TET treatment. Moreover, exogenous cAMP restored the hyphal formation in the TET treatment groups. These results indicate that TET may inhibit the filamentous growth by down-regulating the Ras/cAMP pathway.

Using the *C. elegans* infection model, we revealed the antifungal activity of TET *in vivo*. Since 4 mg/L of TET significantly protected *C. elegans* from *C. albicans* infection *in vivo* but the agent at this concentration could not inhibit the growth of *C. albicans in vitro*, we may attribute the antifungal activity of TET *in vivo* to the inhibitory effect of TET on pathogenic traits. Consistently, TET inhibited yeast-to-hyphae morphological transition, which is the most widely acknowledged pathogenic trait of *C. albicans* [67]. In addition, we tried to use mouse infection model to verify the antifungal activity of TET *in vivo*. The mouse infection model was established by infusion of *C. albicans* via the tail vein. In treatment groups, TET was administered intragastrically at 2 mg/kg, 8 mg/kg or 12 mg/kg for 4 d, and 12 mice was used in each group. TET at 8 mg/kg exhibited protective effect against *C. albicans* infection, but no significance was obtained (*P* > 0.05). Moreover, in 12 mg/kg TET group, mice were thiner and died faster than the control group. Further anatomic study found no food in the stomachs of the mice in the 8 mg/kg and 12 mg/kg TET groups. We used healthy *C. elegans* worms to study the toxicity of TET, and the results indicated that at the final concentration as high as 128 mg/L TET did not exhibit toxicity, which is in accordance with the safe clinical use of TET for silicosis treatment in China since the 1960s[68]. Moreover, it was reported that even when TET was administrated intramuscularly at the dosage of 240 mg, three times daily, it is not toxic to humans[68], [69]. Collectively, TET exhibits low toxicity. Thus, we assumed that the fast death of mice in the above 12 mg/kg TET group was caused by anorexia due to TET intragastrical administration rather than cytotoxicity. Collectively, TET exhibits potential antifungal activity *in vivo*, and further studies should be carried out to improve pharmaceutical preparations and minimize the side effects on mammals.

In conclusion, TET exhibits anti-biofilm effect selectively against *C. albicans*, and the anti-biofilm mechanism may be related to the Ras/cAMP pathway. TET may serve as a tool drug to dissect the difference between *C. albicans* biofilm and other microbial (*C. neoformans*, *A. fumigatus*, *S. aureus* and *P. aeruginosa*) biofilms, and further translational study is required to determine whether the anti-biofilm effect of TET is applicable in clinical settings.

## Materials and Methods

### Strains, culture and agents

*C. albicans* strains and *C. neoformans* strain H99 were routinely grown in YPD (1% yeast extract, 2% peptone and 2% dextrose) liquid medium at 30°C in a shaking incubator [70]. For *A. fumigatus* strain T308073458, conidia were harvested from 3-day-old cultures on Sabouraud dextrose agar plates by flooding the surface of the plates with PBS containing 0.025% (vol/vol) Tween-20 and shaking gently [70]. *S. aureus* was routinely grown in TSB liquid medium at 37°C in a shaking incubator [71]. *P. aeruginosa* was incubated in LB liquid medium at 37°C in a shaking incubator. TET, amphotericin B, ciprofloxacin and penicillin were purchased from Sigma-Aldrich, and fluconazole was purchased from Pfizer inc.

### In vitro biofilm formation assays

To investigate the activities of TET against fungal biofilms, the assays were performed on TET (Sigma, cat. no. T2695) according to the methods that Pierce *et al* described [70] with slight modifications. Briefly, biofilm formation assays were performed in 96-well tissue culture plates (Corning, cat. no. 3599) by seeding 100 µl 1.0×10^6^ cells/ml *C. albicans* cell suspension in RPMI 1640 medium, 200 µl 1.0×10^7^ cells/ml *C. neoformans* cell suspension in DMEM medium (Cellgro, cat. no. 10-013-CV), or 200 µl 1.0×10^5^ cells/ml *A. fumigatus* cell suspension in RPMI 1640 medium (Gibco, Bethesda, MD) respectively, and incubating them statically at 37°C. After 90-min (*C. albicans*) or 4 h (*A. fumigatus* and *C. neoformans*) adhesion, the media were aspirated, non-adherent cells were removed, and fresh medium was added. The plates were further incubated at 37°C for 24∼48 h until formation of mature biofilms. A semiquantitative measure of the formed biofilms was calculated using an XTT reduction assay [72]. To detect the effect of TET and the positive control drug Amphotericin B on the formation of biofilms, different concentrations of the drugs were added to the fresh medium after 90-min/4 h adhesion, incubated at 37°C for 24∼48 h. To detect the effect of the drugs on mature biofilms, the drugs were added after 24-h incubation with the formed mature biofilms at 37°C, and the plates were incubated at 37°C for further 24 h.

To investigate the activities of TET against bacterial biofilms, the assays were conducted as previously described by Malena Sandberg *et al* [71] with slight modifications. Briefly, exponentially growing bacteria were diluted with fresh TSB (for *S. aureus*)/ LB (for *P. aeruginosa*) medium to an OD_600_ of 0.02, then 200 µl cell suspensions were added to flat-bottomed 96-well microplates. Drugs and bacterial suspensions were added simultaneously to the wells of microplates and incubated at 37°C for 18 h. Crystal violet staining of the biofilms was conducted as previously described by Adyary Fallarero *et al* [73]. Briefly, the planktonic suspension was removed from the wells following incubation and the wells were washed with PBS followed by the addition of crystal violet solution (2.3% w/v) and incubated at room temperature for 5 min. The stain was removed and wells were washed twice with PBS. The stained biofilms were then diluted in 96% by volume ethanol and plates were incubated at room temperature for 1 h. The absorbance at 595 nm was measured. Penicillin was used as positive control drug against *S. aureus*, and ciprofloxacin was used as positive control drug against *P. aeruginosa*.

### Measurement of biofilm biomass

Biofilm biomass was measured as described in Nobile *et al* [64] with slight modifications. Sterile silicone squares (1.5×1.5 cm, cut from Cardiovascular Instrument silicone sheets [PR72034-06N, Bentec Medical Inc, United States]) were pretreated with bovine serum (Sigma) overnight and washed with PBS before inoculation. Exponentially growing *C. albicans* cells were diluted to an OD_600_ of 0.2 with Spider medium, and the suspension was added to a sterile 12-well plate with one prepared silicone square in each well. The inoculated plate was incubated at 37°C for 90 min with gentle agitation (150 rpm) until adhesion occurred. To remove non-adherent cells, the squares were washed with 2 ml PBS, and then moved to a fresh 12-well plate containing 2 ml fresh Spider medium. For TET treatment groups, TET was added to the fresh Spider medium. The plate was incubated at 37°C for an additional 60 h at 75 rpm agitation to allow biofilm formation. For dry mass measurements, each biofilm was removed from the substrate by vortexing the silicone square in PBS and then filtering the cell suspension on preweighted filter paper. The filtrate and filter were dried at 75°C overnight and then weighted. The total biomass of each biofilm was calculated by subtracting the weight of the filter paper. The mean dry biomass was calculated from six independent samples. Statistical significance was determined by analysis of variance (ANOVA). Comparison between TET treatment groups and non-treatment group was performed by Student *t* test. A *P* value of less than 0.05 was considered statistically significant.

### Confocal laser scanning microscopy (CLSM) assay

CLSM was performed as described previously [74] to determine the inhibitory effect of TET on biofilm formation. Briefly, plastic disks were inoculated with *C. albicans* statically at 37°C for 90 min to allow adhesion. After removing non-adherent cells, the disk was incubated with fresh RPMI 1640 medium at 37°C for 24 h to allow biofilm formation. For TET treatment groups, TET was added to the fresh RPMI 1640 medium after 90-min adhesion. The disk was then transferred to a new 6-well plate and incubated at 37°C for 45 min in 4 ml PBS containing fluorescent stain FUN-1 (10 µM) (Molecular Probes, Eugene, OR) and concanavalin A-Alexa Fluor 488 conjugate (ConA; 25 mg/L; Molecular Probes). FUN-1 (excitation wavelength 543 nm; emission wavelength 560 nm; long-pass filter) is converted to an orange-red cylindrical intravascular structure by metabolically active cells, while ConA (excitation wavelength 488 nm; emission wavelength 505 nm; long-pass filter) binds to glucose and mannose residues of cell wall polysaccharides and emits green fluorescence. After incubation with the dye, the disk was flipped and *C. albicans* cells were observed under a Leica TCS SP2 CLSM.

### Scanning electron microscopy (SEM) assay

SEM was performed to investigate the ultrastructure of biofilms [75]. Sterile glass disks coated with poly-L-lysine hydrobromide (Sigma, cat. no. P6282) were used to develop *C. albicans* biofilms. The disks were inoculated with *C. albicans* SC5314 statically at 37°C for 90 min to allow adhesion. After removing non-adherent cells, the disks were incubated with fresh RPMI 1640 medium at 37°C for 24 h. For TET treatment groups, TET was added with the fresh RPMI 1640 medium after 90-min adhesion. Biofilms were washed and placed in a fixative consisting of 2% (v/v) glutaraldehyde in 0.15 M sodium cacodylate buffer (pH 7.2) for 2h. The samples were rinsed twice in cacodylate buffer, garnish with 1% osmic acid for 2 h, dehydrated in an ascending ethanol series, treated with hexamethyldisilazane (Polyscience Europe GmbH, Eppelheim, Germany) and dried overnight. The specimens were coated with gold and observed through a Philips XL-30 scanning electron microscope (Philips, The Netherlands) in high vacuum mode [75].

### Cellular surface hydrophobicity (CSH) assay

*C. albicans* CSH was measured by water–hydrocarbon two-phase assay as described previously [76]. In brief, the formed *C. albicans* biofilms were removed from the flask surface with a sterile scraper (Corning, P.R. Mexico) to prepare a cell suspension (OD_600_ = 1.0 in YPD medium). 1.2 ml suspension was pipetted into a clean glass tube and overlaid with 0.3 ml of octane. The mixture was vortexed for 3 min. After the separation of the two phases, OD_600_ of the aqueous phase was determined. OD_600_ for the group without the octane overlay was used as the control. Three repeats were performed for each group. Relative hydrophobicity was obtained as [(OD_600_ of the control minus OD_600_ after octane overlay)/OD_600_ of the control] ×100.

### Time-growth curve assay

Overnight culture of *C. albicans* was diluted with YPD medium to an OD_600_ of 0.01 (about 1.5×10^5^ cells/ml) and divided into 4 bottles. Different concentrations of TET were added to the *C. albicans* suspension. The samples were cultured at 30°C under constant shaking (200 rpm), and cells were counted at the designated time points after culture (0, 3, 6, 9 and 12 h). Three independent experiments were performed [77].

### Antifungal susceptibility test

The MIC values was evaluated for TET in 96-well microtiter plates (Greiner, Germany) as described previously [77], using a broth microdilution protocol modified from the Clinical and Laboratory Standards Institute M27-A3 methods [78], [79]. MIC_50_ was determined as the lowest concentration of the drug that inhibited growth by 50%. MIC_80_ was determined as the lowest concentration of the drug that inhibited growth by 80%.

### Real-time RT-PCR assay

Real-time RT-PCR was used to investigate gene expression changes [80]. Briefly, *C. albicans* SC5314 cells (1.0×10^6^ cells/ml) were added to 80 ml RPMI 1640 medium in 150 mm×25 mm cell culture dishes. The dishes were incubated statically for 90 min to allow initial adhesion, after which the medium was removed and replaced with 80 ml fresh RPMI 1640 medium containing 32 mg/L TET or DMSO as the control. The dishes were then incubated statically at 37°C for further 1 h. *C. albicans* cells were then collected and used for the subsequent RNA extraction. Triplicate independent experiments were conducted on each sample. Total RNA was isolated using Fungal RNAout kit (TIANDS, China). First-strand cDNAs were synthesized using a cDNA synthesis kit (TaKaRa Biotechnology, Dalian, China). Real-time PCR was performed as described previously [80]. Primers are shown in Table S1. Triplicate quantitative real-time PCRs were performed on each sample with SYBR Green II (TaKaRa Biotechnology, Dalian, P.R. China) using ABI 7500 Real-Time PCR system (Applied Biosystems Co, California, USA).

### Determination of cAMP level

Determination of intracellular cAMP level was performed as described previously [81]. *C. albicans* samples were collected as described above for real-time RT-PCR. The *C. albicans* cells were washed once with sterile water and once with MES buffer (10 mM MES [morpholineethanesulfonic acid] containing 0.1 mM EDTA; pH 6). Cells were re-suspended with MES buffer to an OD_600_ of 8, and 500 µl aliquots were taken from the suspension. Samples were transferred to 1.5-ml microcentrifuge tubes containing 0.5 g glass beads and 500 µl 10% trichloroacetic acid, briefly vortexed, and frozen immediately in liquid nitrogen. After centrifugation, trichloroacetic acid was extracted four times with water-saturated ether. The cAMP content was measured using the cAMP Enzyme Immunoassay Kit (Sigma, cat.no.CA200) according to the manufacturer’s instructions.

### Antifungal effect evaluation using a *C. elegans* infection model

*C. elegans*-*C. albicans* infection model was used to evaluate the antifunagl effect of TET. *C. elegans* was infected by *C. albicans* as described previously [82], [83]. Briefly, *C. elegans glp-4; sek-1* adult nematodes were added to the center of *C. albicans* SC5314 lawns on BHI kanamycin (45 mg/L) agar plates and incubated at 25°C for 4 h to allow infections. Worms were washed four times with sterile M9. Sixty worms were then pipetted into each well of six-well tissue culture plates (Corning, USA) containing 2 ml of liquid medium (80% M9, 20% BHI) and kanamycin (45 mg/L). For TET treatment groups, TET was added with a series of concentrations, including 4 mg/L, 8 mg/L, 16 mg/L and 32 mg/L. 32 mg/L FLC treatment group was set as the positive control, and the DMSO solvent group was set as the negative control. Worms were scored daily and dead worms were removed from the assay. Survival was examined by using the Kaplan-Meier method and differences were determined by using the log-rank test (STATA 6; STATA, College Station, TX). A *P* value of<0.05 was considered statistically significant.

### Toxicity evaluation using *C. elegans* worms

To evaluate the toxicity of TET, *C. elegans glp-4; sek-1* adult nematodes were prepared and the assay was performed as described previously [84]. Briefly, the nematodes were moved from *Escherichia coli* OP50 to pathogen-free liquid medium containing 32, 64, and 128 mg/L TET or the solvent DMSO at the same volume. The worms were incubated at 25°C for 2 d and observed daily.

## Supporting Information

Table S1 **Primers used for real-time RT-PCR in this study.**
(DOC)
