# Supplementary material for: Effect of Tetrandrine against Candida albicans Biofilms
Source: PLoS One. 2013 Nov 18;8(11):e79671. doi: 10.1371/journal.pone.0079671 (PMC3832530; doi:10.1371/journal.pone.0079671)
Supplement: Table S1 — Primers used for real-time RT-PCR in this study. (DOC) [file pone.0079671.s001.doc]

| **Table S1**. Primers used for real-time RT-PCR in this study^a^ | |
| --- | --- |
| Primer name | Sequence |
| HWP1-F | CACAGGTAGACGGTCAAGGT |
| HWP1-R | AAGGTTCTTCCTGCTGTTGT |
| ALS1-F | TTGGGTTGGTCCTTAGATGG |
| ALS1-R | ATGATTTCAAAGCGTCGTTC |
| ALS3-F | ACTAAAGGAAACAACGGAAATG |
| ALS3-R | AGAAATGATAGGCGATGAAGC |
| CSH1-F | CTGTCGGTACTATGAGATTG |
| CSH1-R | GATGAATAAACCCAACAACT |
| ECE1-F | GTATTCTTGGCAACATTCC |
| ECE1-R | ACGTCATCATTAGCTCCAT |
| SAP4-F | AGTGTTCTTGCTTTCGCTTTA |
| SAP4-R | TGCCCACATCATTTCTACC |
| SAP5-F | TGTTGACACTGGGTCTTCT |
| SAP5-R | ATACAACTTACCTTTAGCG |
| SAP6-F | CAGTGGCTCTTTAGTTGAA |
| SAP6-R | TACGAGCAATACTTGGAGT |
| UME6-F | CCCATCATCAATCTTACCT |
| UME6-R | CACCACCAATAGAATCAAA |
| HGC1-F | AACCACCACCACCAATGAA |
| HGC1-R | GAAACAGCACGAGAACCAG |
| EED1-F | TAAGTCCCATACAACCAGG |
| EED1-R | TGTGATTACGAGCAGCATT |
| EAP1-F | AGGCAAAGGTGGCTATCAAG |
| EAP1-R | GTGTCAGTCGTGTAGGAGGT |
| IFF4-F | GGCTCCAGTTATACCACAGC |
| IFF4-R | CTATCGGACTTAGCACCATT |
| ALS9-F | GCGTCTCCAGTATCACCCAG |
| ALS9-R | GAGCAATCAATAGTAGCACC |
| HWP2-F | ATTCTACATCTACTCATCCTGCTA |
| HWP2-R | ATGGCTTGTGAGACTTGTGCT |
| BCR1-F | AGTATAATGCTCCTGGTAAGAA |
| BCR1-R | ACGTAAAGGAGGCACGGCATA |
| HSP70-F | CATTTGTTGCCTTCACT |
| HSP70-R | CACTGGTTTACCTGCTT |
| YWP1-F | GCTACTGCTACTGGTGCTA |
| YWP1-R | AACGGTGGTTTCTTGAC |
| RIM8-F | TCTTCTAACGAACACGACCATC |
| RIM8-R | GGCATCAGCAGGCACTC |
| EFG1-F | CGCAACATCTCAAGGAA |
| EFG1-R | TGTGGTGGGATAGGTAC |
| CEK1-F | ACAAGATGGTATAGAGCTC |
| CEK1-R | ATCTCTACCAGGGAATAAA |
| RAS1-F | GGTGGTGTTGGTAAATC |
| RAS1-R | TCTTCTTGTCCAGCAGT |
| ERG11-F | TTGGTGGTGGTAGACAT |
| ERG11-R | CTGCTGGTTCAGTAGGT |
| PGA10-F | TAGCTGCTACCGTGAGT |
| PGA10-R | CAAACAACCAGTATCCC |
| TEC1-F | TCAACAGTCACGAGGAA |
| TEC1-R | TGGCTGGGAGATGC |
| BCY1-F | TTGGAGGATTAGAAGCACTCT |
| BCY1-R | CACATCTGAATCACGACGAA |
| CDC25-F | TACCACGGGACATCAGT |
| CDC25-R | TCAATTACGCTACACGA |
| CPH1-F | AAACCCAGAAGCGTCAT |
| CPH1-R | ACCCAGCAGAAATACCG |
| CPH2-F | GGAGGTATGGCTGCTGT |
| CPH2-R | GGCGATGGGTTAAGAAT |
| CYR1-F | TGAGCCACCAATAGGAC |
| CYR1-R | AACGCATCACCTTCAGT |
| GPR1-F | AATGCTGCTGGTAATGG |
| GPR1-R | TGACTATGTCTCAGGGTA |
| 18S-F | TCTTTCTTGATTTTGTGGGTGG |
| 18S-R | TCGATAGTCCCTCTAAGAAGTG |
| ^a^Abbreviations: F, forward primer; R, reverse primer | |
